# Towards Effective Management Protocols: A Transnational Review of the State-of-the-Art of Coronavirus Disease 2019 (COVID-19) in Pakistan and the United Arab Emirates

**DOI:** 10.7759/cureus.8853

**Published:** 2020-06-26

**Authors:** Maryam Ehtesham, Talal Almas, Absam Akbar, Muhammad Ali Niaz, Noreen Zia

**Affiliations:** 1 Internal Medicine, Royal College of Surgeons in Ireland, Dublin, IRL; 2 Internal Medicine, Aga Khan University, Karachi, PAK; 3 Surgery, Royal College of Surgeons in Ireland, Dublin, IRL

**Keywords:** covid-19, management, pakistan, uae

## Abstract

Coronavirus disease 2019 (COVID-19) has proliferated rapidly in Pakistan, adversely affecting every province. The grave repercussions that the pandemic has elicited in Pakistan have evoked a dire need for drastic measures to be employed at both the governmental and provincial levels. Due to the inequitable appropriation of healthcare resources with respect to the various provinces, however, a stark contrast in terms of morbidity and mortality persists. Furthermore, considering that Pakistani citizens constitute a noteworthy proportion of expatriates residing in the United Arab Emirates (UAE) and the close proximity of the two countries, due consideration of the situation in the UAE is also warranted. We present a transnational review to delineate the current state-of-the-art in Pakistan and the United Arab Emirates and evaluate pragmatic management protocols that remain at the epicenter of a national healthcare conundrum.

## Introduction and background

Coronavirus disease 2019 (COVID-19) was first identified in Wuhan, China, and is characterized by an atypical, or “walking,” pneumonia [1]. Due to the exorbitant proliferation of COVID-19, the novel ailment was termed a pandemic by the World Health Organisation (WHO) [2]. An increasingly cosmopolitan world, and frequent transnational travel, led to the dissemination of the novel virus in countries neighboring China such as South Korea, Japan, Thailand, Sri Lanka, and Iran. In Pakistan, the first two COVID-19 cases were diagnosed on February 26, 2020, in Karachi, and both infected individuals had recently traveled to Iran for a pilgrimage [3]. Within 15 days, the number of infected individuals skyrocketed to 20, all attributed to non-local transmission of the disease and positive for a travel history to Syria, Iran, and/or the UK [4]. The government, as a result of this exponential spike, exerted precautionary measures at the Taftan border that conjoins Iran and subsequently started quarantining all travelers from Iran. By the time these measures were taken, however, as many as 8000 people had already returned from Iran. Shortly thereafter, quarantine camps were instituted in Sukkur and Dera Ghazi Khan for the returning pilgrims [5]. Infected individuals (about 1270 pilgrims returning from Iran, including people from Gilgit-Baltistan and Azad Kashmir) were also shifted to quarantine facilities set up in Multan and Faisalabad [6]. However, poor healthcare facilities, as well as the public’s non-compliance, resulted in further local dissemination of the disease, which diversified to include healthcare professionals [7-8]. Furthermore, the annual Tableeghi Jamaat, invoking the congregation of hundreds of pilgrims, took place in Raiwand despite strict orders of the Punjab government prohibiting it. Subsequently, innumerable attendees tested positive for the new infectious ailment [9]. Similarly, in the United Arab Emirates (UAE), the novel coronavirus was first identified on January 23, 2020, in a family of patients traveling from Wuhan, China, to the UAE on January 16 [10]. On January 23, the grandmother of the family displayed symptoms masquerading the flu, which, upon further work-up and testing, was diagnosed as case 0 of the UAE. Three other members of the family also tested positive; public health was thus notified and consequently instituted measures to promptly curb the novel viral ailment [10]. This review delineates a transnational evaluation of the current state-of-the-art of the COVID-19 pandemic in Pakistan and the UAE. Furthermore, this review draws upon the management protocols that have been employed in both nations, further evaluating their feasibility and curative efficacy.

## Review

Pakistan

On March 18, 2020, Pakistan witnessed the death of the first COVID-19 patient on the day he tested positive in a quarantine facility in Mardan. He had returned from a pilgrimage in Saudi Arabia. By this time, the number of infected individuals had reached 750 [11]. Throughout March, many overseas Pakistani flocked home amidst the fear of a complete lockdown, and these included people arriving from highly infectious areas, including the United Kingdom [12]. By March 24, 2020, Pakistan had reported 990 cases and six deaths [13]. As of May 28, 2020, a total of 508,086 tests have been carried out. Of these, 61,227 positive cases have been reported. Additionally, 1260 deaths have occurred and 20,231 patients have recovered [14]. The cases are well-spread across the different provinces and cities. The provincial distribution of COVID-19 cases in Pakistan, as of May 28, 2020, is tabulated in Table 1 [14].

**Table 1:** Provincial distribution of COVID-19 cases in Pakistan

Province	Number of Cases (as of 28/05/2020) [14]
Sindh	24,206
Punjab	22,037
Khyber Pakhtunkhwa (KP)	8,483
Balochistan	3,616
Islamabad	2,015
Gilgit Baltistan	651
Azad Jammu Kashmir	219

**Figure 1:**
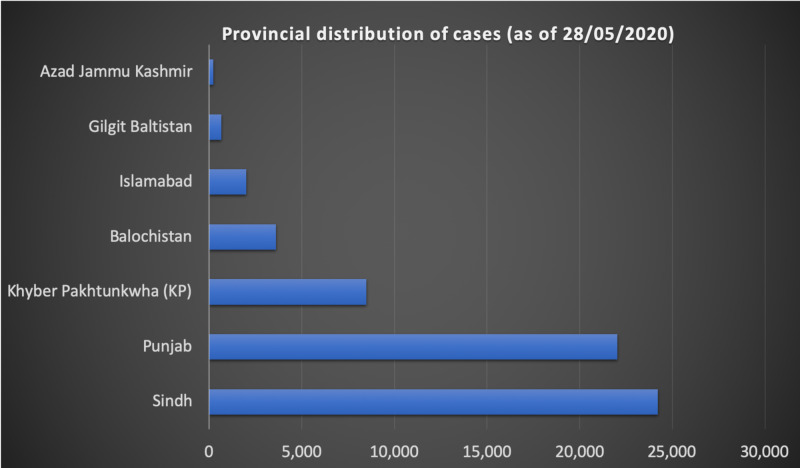
A depiction of the provincial distribution of COVID-19 cases in Pakistan.

Table 1 elucidates the discrepancies in the prevalence of COVID-19 cases amongst various provinces [1]. It can be seen that Sindh and Punjab, the two most populated provinces of Pakistan, boast the highest numbers (24,206 and 22,037, respectively). The other two vastly populated provinces, Khyber Pakhtunkhwa and Balochistan, have significantly lower numbers. Islamabad, the federal capital, also has relatively low numbers but the occurrence is certainly on the rise. Gilgit Baltistan and Azad Jammu & Kashmir, the northern-most regions, have reported merely 651 and 219 cases, respectively. However, there has been an alarming surge in the number of cases since the relaxation of the lockdown, with the number of cases soaring above 30,000 in both Sindh and Punjab as of June 4, 2020 [14]. Figure 1 accentuates the discrepancy in the number of cases detected in the various provinces.

Furthermore, the number of deaths since the emergence of the first case is delineated in Table 2 [15].

**Table 2:** Number of deaths depicted in chronological order

Date	Number of Deaths
26 February	0
17 March	0
18 March	4
25 March	8
01 April	32
08 April	64
15 April	128
22 April	228
29 April	346
06 May	578
13 May	779
20 May	1037
27 May	1276

Currently, the cases are increasing at an exorbitant rate of more than 1000 cases per day as the government transitions toward leniency in the lockdown imposed. Although the economy of the country has suffered directly from the initial lockdown, with many workers losing their jobs (including expatriates), the government is currently implementing a relaxation of the crucial lockdown measures despite the exponential surge in cases. 

A possible explanation for the differential provincial counts

A 2017 population consensus in Pakistan divulged that a majority of the country’s population inhabits Punjab and Sindh, two of the most populous provinces [16-17]. A close-knit familial structure and congested living conditions in these provinces mean that the local transmission of the novel viral ailment in these two provinces has proliferated exorbitantly. In Pakistan, a large number of families, each with multiple members, choose to reside together, which means reduced contact precautions are adhered to. Additionally, inequitable distribution of healthcare budget commensurate with the population numbers is a potent factor that might, in fact, explain the underlying differences. For example, a vast chasm between the population and the number of quarantine facilities that were initially set up persists, explaining the difference in the number of cases detected in each province.

Table 3 elucidates the number of beds available in various quarantine facilities in different provinces [14].

**Table 3:** A provincial breakdown enumerating the total number of beds afforded by quarantine facilities

Region	Total number of beds provided by quarantine facilities
Islamabad	350
Khyber Pakhtunkhwa	2,760
Punjab	10,948
Balochistan	5,897
Sindh	2,100
AJK	530
Gilgit Baltistan	972

Furthermore, Table 4 depicts the number of beds that, as of May 28, 2020, remains unconsumed, thus pointing to the underlying exhaustion of healthcare facilities [14].

**Table 4:** An enumeration of the total bed vacancies in each province

Region/Province	Hospital beds available/vacant
Islamabad	10
Balochistan	534
Khyber Pakhtunkhwa	110
Punjab	955
Sindh	151
Azad Jammu & Kashmir	310
Gilgit Baltistan	126

**Figure 2:**
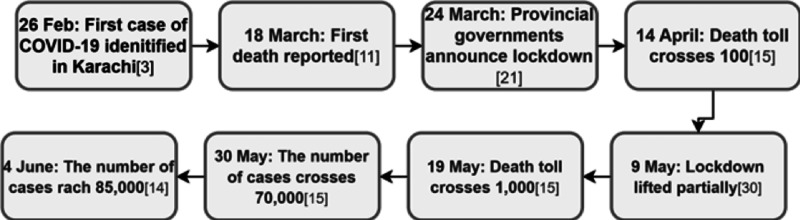
The chronological timeline of coronavirus disease 2019 (COVID-19) proliferation in Pakistan

It is not surprising that the highest number of cases are in the two most densely populated areas, Punjab and Sindh. A statistical comparison divulges that Punjab, with a population about 28 times greater than that of AJK, has merely eight times the quarantine facilities. The situation is much worse in Sindh, which has nearly half the population of Punjab. Sindh, with a population nearly 37 times that of Gilgit Baltistan, was afforded merely twice the number of facilities. This demonstrates the grave lack of facilities and resources in the larger provinces and accounts for the gap in the number of cases. Additionally, the higher numbers in Sindh and Punjab can also be attributed to the fact that many people who tested positive in the earlier stages of the spread were pilgrims who had returned from Iran, which at that time was suffering badly from the viral spread [5]. A lack of adequate quarantine facilities and close proximity of people to each other led to the exponential viral spread in Sindh. Furthermore, at the Taftan border, exhaustion of the quarantine facility due to the influx of pilgrims led to an in-facility proliferation of the virus [18]. This could account for the initial rise in numbers in Balochistan. In Punjab, the Tabhleegi Jamaat, attended by several thousands, was the main reason leading to the widespread proliferation of the disease [9]. Additionally, throughout the course of Ramadan, Taraweeh prayers were carried out at night, invoking the congregation of innumerable people praying in close proximity [19-21]. Figure 2 is a timeline of the chronological order in which COVID-19 has proliferated and thus yielded adverse outcomes, in Pakistan.

**Figure 3:**
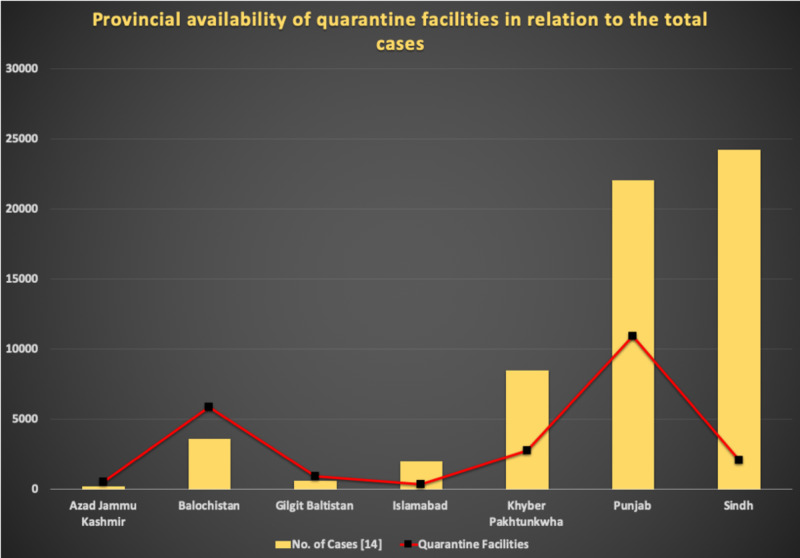
A graphical elucidation of the provincial availability of quarantine facilities in relation to the total number of positive COVID-19 cases

Furthermore, from Figure 3, it is easily discerned that in the largest provinces of Pakistan, namely, Punjab, Sindh, and Khyber Pakhtunkhwa, the exorbitant number of total cases confirmed significantly outweighs the quarantine facilities available. Similarly, in Islamabad, this disparity broadens even further [1]. Islamabad, with 2,015 COVID-19 positive cases, has a quarantine facility consisting of only 350 beds. Despite the fact that Punjab has the highest number of quarantine facilities (10,948 beds) available, these are still grossly insufficient for its astronomical population [14]. Sindh, with the highest number of cases (24,000) initially set up an astonishingly low number of quarantine facilities, with a total hovering at merely 2,100 beds. However, this is still insufficient for a population that borders 50 million [16]. A similar trend can also be seen in Khyber Pakhtunkhwa, where the number of cases (8,400) outweighs the number of beds (2,700) by a significant margin [14]. Figure 3 accentuates the stark contrast that persists in the provincial case tally and the available quarantine facilities. 

Management protocols being employed to thwart COVID-19 proliferation

Punjab

The Punjab government immediately instituted a lockdown, which was implemented on March 24 and has since then been extended throughout the month of May. Schools, colleges, markets, businesses, public transport, and regional motorways were shut down to limit spread [21]. Quarantine centers were set up in Multan for hundreds of pilgrims arriving from Iran [6]. As many as 10,948 quarantine facilities have been set up in the state, Multan being the largest center [14]. The government has permitted the use of the drug Actemra, an interleukin-6 (which plays an important role in the pathogenesis of the disease) inhibitor, in hospitals for intensive care unit (ICU) patients [22]. Furthermore, a 1,000-bed quarantine facility was set up at the Expo Center in Lahore, replete with requisite equipment and kits [23].

Sindh

The Sindh government also responded by imposing a lockdown that commenced on March 24, and all public gathering places were shut down. Pilgrims from Iran were quarantined in Sukkur [7]. Apart from the quarantine facilities that were set up all around the province, with the help of the Pakistan Army, the government established a 10,000-capacity quarantine facility at Karachi’s Expo center, a much-needed step considering the province’s meager hospital quarantine facilities [24]. Additionally, testing supplies, including N-95 masks and kits, were procured from China [25]. A passive immunization trial, using plasma therapy to treat critical COVID patients, has been carried out, boasting a recovery rate of 86% [26].

Khyber Pakhtunkhwa

On March 24, the provincial government announced a lockdown and subsequently installed screening teams at all entry/exit points in the province. Testing kits have since been obtained and dispatched to different cities and several quarantine facilities have been set up, many of these in Peshawar [27]. Paramedics have been given the requisite training and the government has also hired over 1300 doctors to combat the outbreak in the region [28].

Balochistan

A state of emergency was declared on March 24 and was later extended to May. The Taftan border was sealed. Pilgrims have been allowed to return in batches and all of them undergo mandatory quarantine at the Taftan quarantine camp [29]. Funds have been allocated to fight the disease and quarantine centers have been instituted throughout the province [14].

Gilgit Baltistan (GB) & Azad Jammu & Kashmir (AJK)

In both states, quarantine facilities have been set up and the lockdown has been reinforced. Quarantine facilities were set up and testing has since continued. Whilst AJK suspended all forms of transport to limit the spread, the World Health Organization subsequently intervened in GB, providing data management assistance to the state to combat the outbreak [30-31].

Islamabad Capital Territory (ICT)

Similar measures were taken, including extending the lockdown. Additionally, quarantine facilities have been set up in hospitals to cater to the surge in cases [14].

United Arab Emirates

In the United Arab Emirates (UAE), the degree of vigilance rose exponentially in March when cases were being reported daily and asymptomatic individuals were also found to test positive. In the light of the now appreciated ‘pandemic’ status of the outbreak, mass testing measures were set in place to test high-risk areas, including the crowded areas of the various Emirates, with an increased emphasis on occupations that evoked frequent human contact such as taxi drivers. Curfews were set nationwide and individuals had to obtain permits to go grocery shopping as the National Sterilization Program commenced, ensuring deep cleaning and sanitation of every neighborhood in every Emirate across the country. March 6 marked the first two deaths due to COVID-19 in the UAE. At this stage, the total number of cases remained at a mere 140 [32-33]. As testing measures were rigidly enforced, an average of hundreds of cases began being detected every day. By April 27, the UAE had exhausted 1 million tests in their quest to prevent the spread of the virus [33-34]. The holy month of Ramadan saw a degree of lenience in regards to curfew regulations nationwide. Despite the lockdown being reinforced on the days preceding and including Eid, the daily number of cases had already reached the late hundreds at that point in time. As of May 29th, 638 cases were reported on the day, adding to the total number of 33,170 cases recorded [32-36].

Strategies for curbing and managing COVID-19 proliferation

Curfew

All individuals, excluding individuals in certain sectors, were made to work from home. As a result, schools, universities, and workplaces are to remain remote until September 2020. All entertainment activities were suspended and restaurants were made to offer delivery services only. Additionally, centers for the registration of marriages and divorce alike were suspended on April 8. Curfew regulations were smoothly introduced to residents as the aforementioned National Sterilisation Program commenced on March 27 [32-33]. Curfew measures involved daily message reminders of curfew hours along with strict monitoring on the roads of every Emirate, beginning 30 minutes prior to the curfew time. Fines of AED 10,000 were set in place for curfew violations. As of May 30, the curfew measures were once again relaxed slightly and the Emirate of Dubai saw the commencement of various entertainment facilities, including malls, cinemas, and gyms, with a restriction imposed on those under 12 and over 65 years of age. These facilities were only to remain open on strict adherence to appropriate social distancing measures.

Health

Heavy emphasis was placed on door-to-door testing in the crowded areas of apartment complexes in Dubai. High-risk groups were aptly managed in the speedy tracing of contacts of reported cases. Similarly, on May 22, the Emirate of Sharjah tested over 90,000 employees in 31 work accommodations across the Emirate. By employing a generalized national approach to testing and treating the virus, the UAE was able to keep track of all cases and prevent the unintentional spread of the virus with their designated centers for coronavirus testing and treatment in each Emirate. These centers were run by doctors and interns in addition to medic and non-medic volunteers from the general population who chose to pitch into the efforts of remitting the outbreak.

Sterilization Program

The UAE began a large scale sterilization campaign wherein as many as 90 roads were disinfected in early day hours [32]. Along with this, a large-scale drone sterilization campaign was also carried out to disinfect several buildings and public spaces, to limit the viral spread [33]. A mobile drive-through test center was also set up [35].

Travel

Beginning on March 22, UAE stopped its international flight operations, apart from a few selected destinations, as a measure to limit traveling [35]. Furthermore, public transport services such as the Dubai Metro were also suspended to ensure that passengers traveling in close proximity do not serve as catalysts in proliferating the infection [36].

## Conclusions

Coronavirus disease 2019 (COVID-19) has resulted in wide-spread, grave morbidity and mortality in both Pakistan and the United Arab Emirates. In Pakistan, a failure to apportion healthcare resources optimally has resulted in provincial disparities, which are reflected by the burgeoning mortality rates in Punjab and Sindh, the two most populous provinces. While the United Arab Emirates is situated in Pakistan's close geographical proximity, the dispensable resources it fosters in combatting the pandemic hover beyond those accessible to Pakistan. Thus, there is an unmet need for healthcare reforms to be instituted both at the provincial and governmental levels in Pakistan. Additionally, a transnational collaboration between Pakistan and the United Arab Emirates can ameliorate the current state-of-the-art in both the nations, effectively affording the ability to thwart further viral proliferation.
